# Supplementary material for: Machine Learning Models for Prediction of Severe Pneumocystis carinii Pneumonia after Kidney Transplantation: A Single-Center Retrospective Study
Source: Diagnostics (Basel). 2023 Aug 23;13(17):2735. doi: 10.3390/diagnostics13172735 (PMC10486565; doi:10.3390/diagnostics13172735)
Supplement: Supplementary file 1 [file diagnostics-13-02735-s001.zip › diagnostics-2546988-supplementary.pdf]

Table S1. Multicollinearity analysis of model variables

Variable	VIF
PCT	1.502
Hb	1.374
ALB	1.318
Progressive dyspnea	1.313
CRP	1.106

VIF:Variance Inflation Factor; CRP: C-reactive protein; PCT: procalcitonin; Hb: Hemoglobin; ALB: albumin.

Table S2. Tuning parameters of the predictive models

Classification model	Hyperparameter	Define Hyperparameter Ranges	Optimum value
LR	C	0.01~10	10
	max_iter	10~500	100
	penalty	l2/none	l2
	tol	1.00E-09~0.1	1.00E-06
XGB	objective	binary:logistic/binary:logitraw/reg:linear/reg:logistic	binary:logistic
	learning_rate	0.001~2	0.1
	max_depth	1~20	8
	min_child_weight	1~20	2
	reg_lambda	0.001~5	1
RF	criterion	gini/entropy	gini
	max_depth	1~20	None
	min_impurity_decrease	0~20	0
	n_estimators	1~200	100
SVM	C	0.01~10	1
	kernel	rbf/linear/poly/sigmoid	rbf
	tol	1.00E-09~0.1	0.1
LGBM	boosting_type	gbdt/dart/goss	gbdt
	learning_rate	0.001~2	0.001
	max_depth	-1~20	1
	n_estimators	1~200	50
	num_leaves	1~200	5
KNN	n_neighbors	1~20	5
	weights	uniform/distance	uniform

Using five-fold cross-validation in the training set, the AUC in the internal validation set is maximised by grid-tuning parameters to determine the optimal hyperparameters.

Table S3.Summary of the specific performance of the six machine algorithm models in the internal validation set.

	AUC(95%CI)	Accuracy(95%CI)	Sensitivity(95%CI)	Specificity(95%CI)	PPV(95%CI)	NPV(95%CI)	F1_Score(95%CI)
RF	0.951(NaN-NaN)	0.896(0.839-0.952)	0.920(0.847-0.993)	0.933(0.846-1.020)	0.920(0.847-0.993)	0.882(0.809-0.955)	0.917(0.865-0.968)
LR	0.948(NaN-NaN)	0.917(0.865-0.969)	0.920(0.847-0.993)	0.933(0.846-1.020)	0.944(0.871-1.016)	0.901(0.847-0.955)	0.929(0.871-0.988)

XGB	0.906(0.780-1.000)	0.814(0.741-0.886)	0.940(0.862-1.018)	0.831(0.775-0.887)	0.818(0.774-0.862)	0.836(0.718-0.953)	0.874(0.817-0.932)
SVM	0.960(NaN-NaN)	0.886(0.827-0.945)	0.920(0.806-1.034)	0.958(0.907-1.009)	0.905(0.825-0.985)	0.884(0.812-0.956)	0.911(0.818-1.003)
KNN	0.967(0.905-1.000)	0.834(0.731-0.937)	0.936(0.884-0.987)	0.936(0.849-1.022)	0.978(0.934-1.021)	0.777(0.654-0.900)	0.954(0.932-0.977)
LGBM	0.814(0.634-0.976)	0.563(0.451-0.676)	0.836(0.720-0.951)	0.809(0.724-0.894)	NaN(NaN-NaN)	0.577(0.438-0.715)	NaN(NaN-NaN)

RF: Random Forest; LR: Logistic Regression; XGB: eXtreme Gradient Boosting; SVM: Support Vector Machine; KNN: K-Nearest Neighbor; LGBM: light gradient boosting machine; AUC: area under the curve; PPV: PositivePredictive Value; NPV: Negative predictive value
